# In this month’s Bulletin

**DOI:** 10.2471/BLT.24.000724

**Published:** 2024-07-01

In the editorial section, Irene Papanicolas et al. (458) introduce this special theme issue on health system performance assessment. 

Adèle Sulcas (461–462) reports on health system performance assessment after the COVID-19 pandemic. Githinji Gitahi talks to Gary Humphreys (463–464) about how health system assessment contributes towards achieving universal health coverage. 

## Argentina, Colombia, Ethiopia, Greece, India, Italy, Kenya, Lao People’s Democratic Republic, Mexico, Nigeria, Peru, Republic of Korea, South Africa, United Kingdom of Great Britain and Northern Ireland, United States of America, Uruguay

### Does the health sector meet expectations?

Margaret E Kruk et al. (486–497) administer the People’s Voice Survey.

## Bangladesh, India, Nepal, Pakistan, Sri Lanka

### Primary health care measurement

Neha Purohit et al. (476–485) assess a WHO/UNICEF framework.

## Belgium, Croatia, Czechia, Estonia, Ireland, Italy

### Measuring resilience

Milena Vainieri et al. (498–508) examine performance assessments.

## Ethiopia

### Primary care dashboards

Catherine Arsenault et al. (465–475) surface routine data. 

## India

### Provision of hospital care

Liana Woskie et al. (509–520) study patient satisfaction.

## Oman

### Improving health system performance 

Taavi Lai et al. (533–537) describe an assessment and reform cycle.

## Global

### Opportunities and limits of evaluation

Kevin Croke et al. (538–540) list new options.

### Assessment and public health 

Jochen O Mierau et al. (541–543) make a case for systems improvement.

### Health system characteristics

Ruth Waitzberg et al. (547–549) detail the analysis needed before assessment. 

### Multisectoral interventions and health system performance

I Nyoman Sutarsa et al. (521–532) review the evidence.

### Meeting societal goals

Rachel Greenley et al. (544–546) note health system contributions.

### Using policy questions

Irene Papanicolas et al. (550–552) propose a basis for performance comparisons.

